# Preventive Effects against Retinal Degeneration by *Centella asiatica* Extract (CA-HE50) and Asiaticoside through Apoptosis Suppression by the Nrf2/HO-1 Signaling Pathway

**DOI:** 10.3390/antiox10040613

**Published:** 2021-04-16

**Authors:** Dae-Won Park, Yeong-Geun Lee, Yong-Joon Jeong, Hyelin Jeon, Se-Chan Kang

**Affiliations:** 1 Department of Oriental Medicine Biotechnology, College of Life Sciences, Kyung Hee University, Yongin 17104, Gyeonggi-do, Korea; dw@nmr.kr (D.-W.P.); lyg629@nate.com (Y.-G.L.); 2 Research Institute, Genencell Co., Ltd., Yongin 16950, Gyeonggi-do, Korea; jeyoon@genencell.co.kr

**Keywords:** *Centella asiatica*, asiaticoside, age-related macular degeneration, cytoprotective effect, Nrf2/HO-1 antioxidant signaling pathway, cell cycle, apoptosis

## Abstract

Age-related macular degeneration (AMD) is caused by the chronic and gradual oxidative degeneration of the retina. Unfortunately, the general purpose of current treatments is to slow AMD progression, as the retina cannot be restored to its pre-AMD condition. We aimed to identify natural products that can be potential treatments that prevent AMD and can delay the development of late-AMD and selected *Centella asiatica* extract (CA-HE50), which shows excellent efficacy in cytoprotection. In animal experiments using *N*-methyl-*N*-nitrosourea (MNU), CA-HE50 dramatically increased the thickness of photoreceptors and the outer nuclear layer (ONL) and the number of nuclei in the ONL (*p* < 0.05). Using retinal epithelial ARPE-19 cells showed that CA-HE50 inhibited apoptosis through inhibition of the intrinsic apoptosis signaling pathway and cell cycle regulation (*p* < 0.05). The anti-apoptotic efficacy was confirmed to be due to activation of the Nrf2/HO-1 antioxidation pathway (*p* < 0.05). These results were also observed with asiaticoside, a functional substance of CA-HE50. In addition, the accumulation of oxidized-*N*-retinylidene-*N*-retinylethanolamine (A2E), which induces AMD, was inhibited by CA-HE50, resulting in increased ARPE-19 cell viability (*p* < 0.05). This study demonstrates that CA-HE50 is worth further research and human application tests, to develop it as a raw material for treatment or dietary supplement for the prevention of AMD.

## 1. Introduction

The increase in human life expectancy naturally leads to an increase in the number of patients with age-related diseases such as cancer, cardiovascular disease, diabetes, and joint, tooth, and eye diseases. Older age can lead to eye diseases such as cataracts, glaucoma, and age-related macular degeneration (AMD). AMD, which is caused by the chronic and gradual degeneration of the retina, is the third leading cause of blindness worldwide and a major cause of vision loss in Western society [1,2,3]. Risk factors for developing AMD generally include patient characteristics (age, gender, social class, and ethnic group), vascular factors (cardiovascular disease, dietary fat intake, smoking, alcohol consumption, and estrogen), oxidative processes, and genetic factors [4]. Clinically, AMD is divided into two types: dry AMD (non-neovascular, non-exudative) and wet AMD (neovascular, exudative). In addition, AMD can be divided into early, intermediate, and late stages. The early and mid-stages are mostly dry AMD, which is associated with drusen and pigment changes that cause minimal visual symptoms. Late AMD includes dry AMD characterized by geographic atrophy disease in the macular region and wet AMD characterized by choroidal neovascularization, which can cause severe visual impairment [5,6]. Treatment of AMD depends on the situation at the time of diagnosis. However, there is currently no treatment that can reverse the effects of AMD, so treatment generally aims to slow AMD progression [7].

During cellular metabolism, living organisms produce reactive oxygen species (ROS) from molecular oxygen. ROS levels are rigidly regulated to maintain cellular homeostasis [8]. The level of ROS is regulated by an antioxidant system composed of enzyme and non-enzyme molecules. Non-enzymatic antioxidants are low-molecular-weight compounds that include vitamins C and E, beta carotene, and glutathione. Superoxide dismutase (SOD), catalase, and glutathione metabolism, which are responsible for most of the enzymatic antioxidant defenses, are regulated at the transcriptional level by the transcription factor nuclear factor erythroid 2-related factor 2 (Nrf2) [9]. Previous studies have shown that redox homeostasis in the retinal pigment epithelium is dependent on the activation of Nrf2 [10,11,12]. Lutein, a physiological pigment, activates Nrf2 in ARPE-19 cells (human retinal pigment epithelial cell) [13], and mesozeaxanthin protects against chronic and cumulative eye damage by reducing oxidative stress [14]. For this reason, lutein and zeaxanthin are considered substances that prevent AMD and are marketed as dietary supplements for eye health. Supplements containing antioxidants and minerals are thus consumed worldwide as a preventive measure against eye diseases [15,16]. However, β-carotene, retinol, and lutein supplements cannot be used long-term, as studies have shown that the risk of total lung cancer and histologic cell type cancer significantly increased with these supplements [17]. Therefore, the identification of novel treatments for AMD prevention without side-effects and for long-term use is urgently required.

*Centella asiatica*, also called Gotu Kola or pennywort, has been used in traditional medicine in India, China, Korea, and Southeast Asia to treat a variety of diseases, including wound healing [18]. In our previous study, we confirmed the cytoprotective effect of CA-HE50, which is an extract of *C. asiatica*, in ARPE-19 cells and mouse models. Our study demonstrated that CA-HE50 protects ARPE-19 cells against the induction of cytotoxicity by CoCl_2_ and oxidized-*N*-retinyl-*N*-retinylidene ethanolamine (A2E) and has the effect of inhibiting macular degeneration in animals treated with *N*-methyl-*N*-nitrosourea (MNU) [19]. However, the exact mechanism has remained unknown. Therefore, the aim of the present study was to evaluate the cytoprotective effect of CA-HE50 in ARPE-19 cells and C57BL/6 mouse macular tissues and to clarify the underlying mechanism. In this study, we confirmed the inhibition of A2E accumulation and macular degeneration by the Nrf2-HO-1 antioxidation signaling pathway as a pathway to prevent macular degeneration by CA-HE50. In addition, the effects of CA-HE50 effects on cleavage inhibition of caspase-3 and PARP and cell cycle arrest were studied. Through this study, we verified the cytoprotective efficacy and mechanism of CA-HE50 against macular damage. These findings confirm the potential of developing CA-HE50 as a natural raw material for preventing macular degeneration and demonstrate that CA-HE50 is worth further study such as in human application tests.

## 2. Materials and Methods

### 2.1. Chemicals

All chemicals used in this work were purchased from commercial sources. All solvents were distilled via standard methods prior to use. Asiaticoside, *N*-retinylidene-*N*-retinylethanolamine (A2E), and 3-(4,5-dimethylathiazol-2-yl)-2,5-diphenyltetrazolium bromide (MTT) were purchased from Sigma-Aldrich (St. Louis, MO, USA). MNU was purchased from Spectrum Chemical (New Brunswick, NJ, USA).

### 2.2. Preparation of Samples

#### 2.2.1. Preparation of *C. asiatica* Extract (CA-HE50)

*C. asiatica* was purchased from a plantation in Hapcheon-gun (Gyeongsangnam-do, Korea) in August 2017 and was identified by Professor Kang from Kyung Hee University (Yongin, Gyeonggi-do, Korea). *C. asiatica* was dried while avoiding sunlight. The sample was precipitated in 50% ethanol for 8 h at 80 °C and was concentrated to 20–25 Brix at reduced pressure and 65 °C in a rotary evaporator. The extract was spray-dried to obtain powder and stored at −20 °C until use. We refer to *C. asiatica* 50% ethanol extract as “CA-HE50” throughout this research paper.

#### 2.2.2. HPLC Analysis of Asiaticoside in CA-HE50

CA-HE50 was analyzed by high-performance liquid chromatography (HPLC) using an Agilent 1260 Infinity separation module coupled to a DAD detector (Agilent Technologies, Santa Clara, CA, USA), a Cadenza C18 column (250 mm × 4.6 mm, 3 μm) (IMTAKT, Portland, OR, USA), and a flow rate of 1.0 mL/min. The column was placed in a column oven at 40 °C. The gradient eluted consisted of distilled water (A) and acetonitrile (B). The mobile phase was used under binary linear-gradient conditions as follows: ratio of mobile phase A and B were changed after 0 (90:10, *v/v*), 10 (80:20, *v/v*), 40 (73:27, *v/v*), 45 (80:20, *v/v*), 51 (20:80, *v/v*), and 55 min (90:10, *v/v*). The injection volume was 10 μL and UV detection was performed at 206 nm. Confirmation of CA-HE50 compound content was performed using the external standard method and asiaticoside (ChemFaces, Wuhan, Hubei, China) as the standard stock solution (15.625, 31.25, 62.5, 125, 250, and 500 μg/mL). Peaks were identified by comparing their retention time and UV-vis spectra with the reference compound, and data were quantitated using the corresponding curves of the reference compound as standards. HPLC-grade acetonitrile, distilled water (Fisher Scientific, Waltham, MA, USA), and methanol (Duksan, Ansan-si, Gyeonggi-do, Korea) were used.

### 2.3. In Vivo Analysis

#### 2.3.1. Animal Care and Experimental Design

Seven-week-old-male C57BL/6 mice were purchased from NARA Biotech (Seoul, Korea) and acclimated for one week before the experiment. Mice were housed in controlled environments of temperature (23 ± 5 °C), humidity (55 ± 6%) under 12 h light/dark cycle, and ventilation of 10–12 times per h. Animal experiments were performed in accordance with the current ethical regulations for animal care and use at Kyung Hee University (KHGASP-20-293). The mice weighed 23 ± 2 g and were randomly divided into five groups: normal control (NC); MNU (vehicle control); and MNU+CA-HE50 at three concentrations (50, 100, and 200 mg/kg) (MNU+CA-HE50). After an acclimation period, mice were administered CA-HE50 dissolved in saline orally for seven days. MNU (Spectrum Chemical) dissolved in physiological saline containing 0.05% acetic acid was intraperitoneally administered at a concentration of 50 mg/kg, and mice were sacrificed 24 h later.

#### 2.3.2. Fixation of Eyes and Hematoxylin and Eosin (H&E) Staining

At the time of sacrifice, eyes were washed, collected, and fixed immediately in 4% paraformaldehyde (Sigma-Aldrich) for 4 h. The eyeball was dissected at the equator and the anterior eye and vitreous, including the lens, were removed. The tissues were left in 10% and 20% sucrose solution for 1 h and 30% sucrose solution for 12 h. After embedding tissues in optical cutting temperature (OCT, SaKura Finetek, Torrance, CA, USA) solution, 5 µm thick tissue sections were prepared and stained with H&E (Sigma-Aldrich).

#### 2.3.3. Western Blot Analysis of Ocular Tissue

At the time of sacrifice, left eye was washed, collected, and lysed using protein extraction solution (iNtRON, Seoul, Korea). The obtained protein was quantified using a BCA protein assay kit (BIO-RAD, Hercules, CA, USA). The subsequent procedure was used slightly modified from the experimental method of the previous study [19]. Primary antibodies against caspase-3, Nrf2, and HO-1 were purchased from Santa Cruz Biotechnology (CA, USA) and antibody against β-actin was obtained from Cell Signaling Technology (Beverly, MA, USA). The secondary antibodies were horseradish-peroxide-conjugated anti-mouse and anti-rabbit antibodies (Cell Signaling Technology). The protein bands were developed by ATTD Corporation (Tokyo, Japan).

### 2.4. In Vitro Analysis

#### 2.4.1. Cell Culture

ARPE-19 human retinal pigment epithelium cells were obtained from American Type Culture Collection (ATCC, Manassas, VA, USA). ARPE-19 cells were cultured in Dulbecco’s modified Eagle’s medium/Nutrient Mixture F12 (DMEM/F12 containing glucose concentration of 17.5 mM, Gibco, Grand Island, NY, USA) supplemented with 10% fetal bovine serum (FBS, Hyclone, South Logan, UT, USA), and 1% penicillin/streptomycin in a 37 °C incubator with a 5% CO_2_ atmosphere. Cells were sub-cultured twice each week.

#### 2.4.2. MTT Assays

ARPE-19 cells were seeded at a density of 3 × 10^4^ cells/well in 96-well plates. After incubating overnight, the cells were pretreated with CA-HE50 for 2 h and then treated with MNU (200 μg/mL) for 24 h. After 24 h, 10 μL of MTT (5 mg/mL) solution were added to each well and samples were incubated for 4 h. The supernatant of each well was removed and 100 μL of DMSO were added. The absorbance of the dissolved formazan was measured at 550 nm using the infinite M200 spectrophotometer (TECAN, Männedorf, Switzerland).

#### 2.4.3. Western Blot Analysis

ARPE-19 cells were lysed using radioimmunoprecipitation (RIPA) buffer (ThermoFisher Scientific, Waltham, MA, USA) for 1 h on ice. After centrifugation at 10,000 rpm for 10 min at 4 °C, whole-cell lysates were collected. Equal amounts of protein (40 μg), measured by standard curves using bovine serum albumin (BSA, Sigma-Aldrich), were boiled for 5 min. The proteins were separated by 12% sodium dodecyl sulfate–polyacrylamide gel electrophoresis (SDS-PAGE) and transferred to nitrocellulose membranes. Membranes were blocked with TBS-T buffer (0.1% Tween-20 containing Tris-buffered saline (TBS, Bio-Rad)) containing 5% skim milk and then incubated with primary antibodies overnight at 4 °C. Primary antibodies against caspase-3/-8/-9, PARP, Nrf2, HO-1, p21, cdk2, and cyclin A were obtained from Santa Cruz Biotechnology and antibody against β-actin was purchased from Cell Signaling Technology. The membranes were washed in TBS-T buffer five times and then incubated with secondary antibodies for 2 h at room temperature. The secondary antibodies were horseradish-peroxide-conjugated anti-mouse and anti-rabbit antibodies (Cell Signaling). The protein bands were developed and analyzed by a chemiluminescence imaging system provided by ATTD Corporation.

#### 2.4.4. Cell Cycle Analysis

Cell cycle distributions were analyzed using a Tali^®^ image-based cytometer (ThermoFisher Scientific) according to the manufacturer’s protocol. ARPE-19 cells were pre-treated with CA-HE50 for 2 h and then treated with MNU for 24 h. After harvesting, cells were slowly resuspended and incubated overnight with 70% cold ethanol at −20 °C. Cells were washed with PBS and stained with Tali^®^ Cell Cycle Solution for 30 min in the dark. Cells were analyzed using the Tali^®^ image-based cytometer.

### 2.5. Statistical Analysis

Results were expressed as mean ± standard deviation (SD) for each group. Statistical analysis was carried out using one-way analysis of variance (ANOVA) followed by Tukey’s test in SPSS 12.1K (IBM, Chicago, IL, USA). A *p*-value of less than 0.05 was judged as significant.

## 3. Results

### 3.1. Protective Effect of CA-HE50 against MNU-Induced Retinal Degeneration

**Figure 1:**
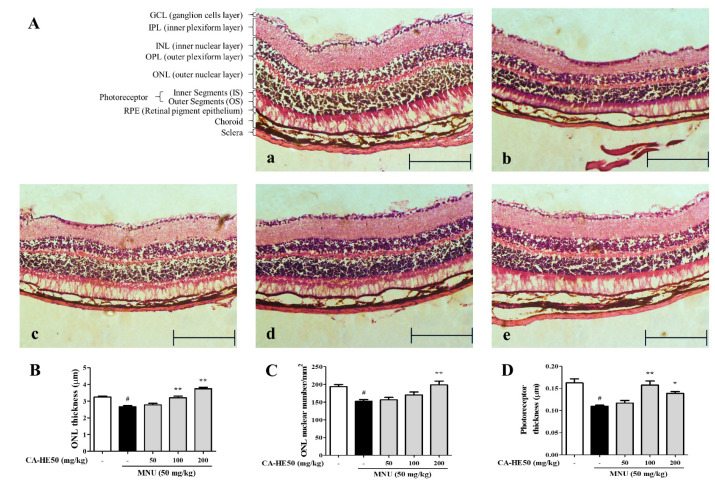
Histopathology images of retinas with H&E staining from five groups of mice 24 h post-injection of MNU. (**A**) Images of H&E staining of a retinal section from mice: (**a**) representative image of H&E staining of a retinal section from control mice (not injected with MNU); (**b**) representative image of H&E staining of a retinal section from MNU/vehicle mice (MNU-injected and saline administered); (**c**) representative of H&E staining of a retinal section from low-dose CA-HE50–administered mice (50 mg/kg CA-HE50 for seven days and then injected with MNU); (**d**) representative image of H&E staining of a retinal section from middle-dose CA-HE50–administered mice (100 mg/kg CA-HE50 for seven days and then injected with MNU); and (**e**) representative image of H&E staining of a retinal section from high-dose CA-HE50–administered mice (200 mg/kg CA-HE50 for seven days and then injected with MNU). Data are representative of six independent animals. (**B**) ONL thickness. (**C**) ONL nuclear numbers. (**D**) photoreceptor thickness. ^#^ *p* < 0.05 vs. normal control group. * *p* < 0.05 and ** *p* < 0.01 vs. MNU-treated group. Abbreviations: MNU, *N*-methyl-*N*-nitrosourea; GCL, ganglion cell layer; IPL, inner plexiform layer; INL, inner nuclear layer; OPL, outer plexiform layer; ONL, outer nuclear layer; IS, inner segments; OS, outer segments; RPE, retinal pigment epithelium.

MNU is acts as a carcinogenic, mutagenic, teratogenic, and alkylating agent and has been studied for use as a cancer chemotherapy agent in mutagenic and genetic studies. For this reason, MNU is used to induce retinal degeneration in animal models [20]. We evaluated the protective effect of CA-HE50 on retinal cells in mice administered MNU. As shown in Figure 1A, all cells of the ocular tissue were damaged by MNU and the tissue thickness was dramatically thinner compared with the control. In particular, the outer nuclear layer (ONL) thickness and photoreceptor thickness changed remarkably with MNU treatment. In mice treated with CA-HE50, the thickness of the ONL, which was reduced by MNU, increased significantly (Figure 1B). We counted the number of nuclei in the ONL per unit area and found that the number of cells decreased by MNU and was recovered by CA-HE50 (Figure 1C). In addition, the photoreceptor thickness was decreased by MNU and recovered by CA-HE50 (Figure 1D). All results were statistically significant (*p* < 0.05), and CA-HE50 dose-dependent results were obtained for all parameters except photoreceptor thickness (Figure 1D).

**Figure 2:**
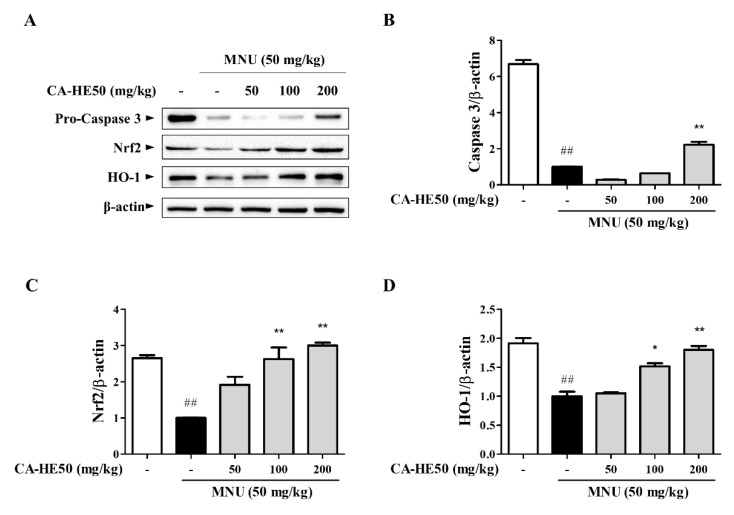
Protein expression from five groups of mice 24 h post-injection of MNU. (**A**) Images of Western blot analysis for expression of proteins associated with retinal damage with MNU. CA-HE50 efficacy results on: (**B**) pro-caspase-3 protein expression; (**C**) Nrf2 protein expression; and (**D**) HO-1 protein expression. ^##^ *p* < 0.01 vs. normal control group. * *p* < 0.05 and ** *p* < 0.01 vs. MNU-treated group. Abbreviations: MNU, *N*-methyl-*N*-nitrosourea; Nrf2, nuclear factor erythroid 2; HO-1, heme oxygenase-1.

Meanwhile, the effect on cell death by MNU and cell protection by CA-HE50 was confirmed through Western blot analysis using eye tissue. As shown in Figure 2A,B, the expression level of caspase-3, which is well known to induce apoptosis, was confirmed, and the expression of pro-form caspase-3 was confirmed to increase in a concentration-dependent manner with CA-HE50 (*p* < 0.01). In addition, as a result of confirming the expression of Nrf2/HO-1, an antioxidant signaling pathway, CA-HE50 significantly increased the expression of Nrf2 and HO-1, and the result was concentration dependent (*p* < 0.05). In particular, in the mice given 200 mg/kg of CA-HE50, the expression levels of the two biomarkers were similar to those of the normal control group (Figure 2A–D).

### 3.2. CA-HE50 Inhibits MNU-Induced Apoptosis in ARPE-19 Cells

**Figure 3:**
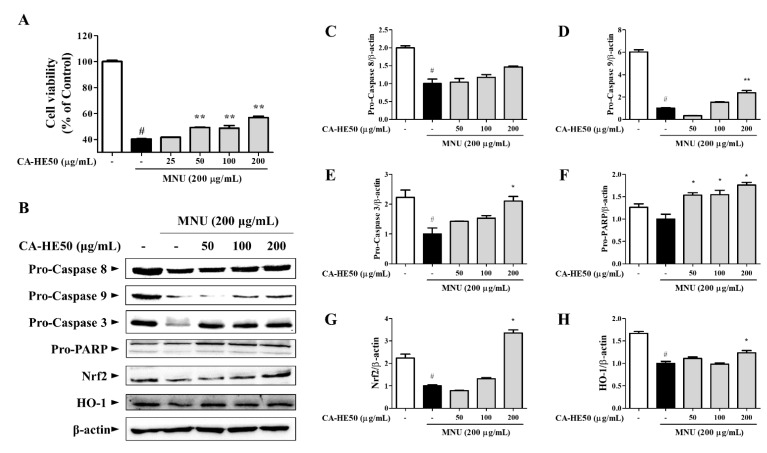
Antiapoptotic efficacy of CA-HE50 through the activated Nrf2/HO-1 antioxidant signaling pathway. (**A**) Cytoprotective effect of CA-HE50 against MNU. (**B**) Western blot analysis for expression of proteins associated with retinal damage with MNU. CA-HE50 efficacy results on: (**C**) pro-caspase-8 protein expression; (**D**) pro-caspase-9 protein expression; (**E**) pro-caspase-3 protein expression; (**F**) pro-PARP protein expression; (**G**) Nrf2 protein expression; and (**H**) HO-1 protein expression. ^#^ *p* < 0.05 vs. normal control group. * *p* < 0.05 and ** *p* < 0.01 vs. MNU-treated group. Abbreviations: MNU, *N*-methyl-*N*-nitrosourea; PARP, poly (ADP-ribose) polymerase; Nrf2, nuclear factor erythroid 2; HO-1, heme oxygenase-1.

MNU showed strong cytotoxicity in ARPE-19 cells, and CA-HE50 increased the viability of ARPE-19 cells in a concentration-dependent manner (Figure 3A) (*p* < 0.05). We further evaluated the expression of proteins related to apoptosis by Western blot analysis to determine the anti-apoptotic efficacy of CA-HE50 (Figure 3B). MNU was found to induce apoptosis by activating PARP through the caspase-9/caspase-3 pathway. CA-HE50 inhibited caspase activation by increasing the pro-form expressions of caspase-9 and caspase-3. These results were concentration-dependent and statistically significant (*p* < 0.05) (Figure 3D–F). However, CA-HE50 did not elicit notable changes in the increase of caspase-8 pro-form, but it tended to inhibit caspase-8 activation (Figure 3C). Significant activation of the Nrf2/HO-1 antioxidant pathway was observed only at a high concentration of CA-HE50, but there was a concentration-dependent tendency (Figure 3G,H) (*p* < 0.05).

### 3.3. CA-HE50 Inhibits Apoptosis through Inhibition of MNU-Mediated S Phase Arrest

**Figure 4:**
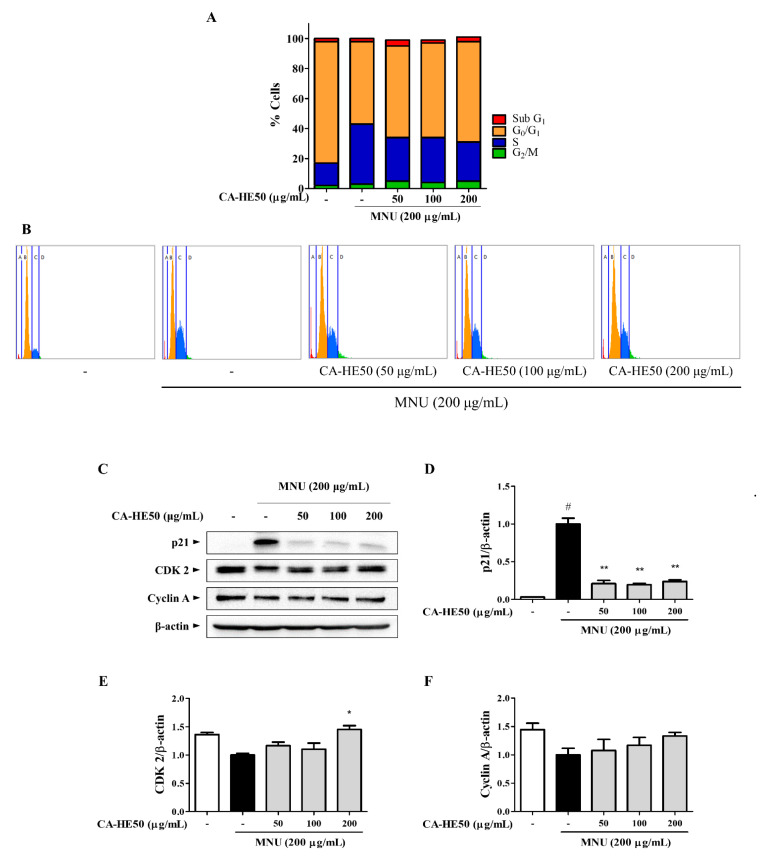
CA-HE50 suppressed MNU-induced S phase arrest of cells by inhibiting p21 expression. (**A**,**B**) Effect of MNU and CA-HE50 on cell cycle distribution. (**C**) effect of CA-HE50 on cell cycle–related protein expressions. CA-HE50 efficacy results on: (**D**) p21 protein expression; (**E**) CDK2 protein expression; and (**F**) cyclin A protein expression. ^#^ *p* < 0.05 vs. normal control group. * *p* < 0.05 and ** *p* < 0.01 vs. MNU-treated group. Abbreviations: MNU, *N*-methyl-*N*-nitrosourea; CDK2, cyclin-dependent kinase 2.

We next conducted experiments to determine the effect of MNU and CA-HE50 on the cell cycle. In cells treated with MNU, which causes cytotoxicity, the cell cycle was arrested in the S phase (Figure 4A,B). These results were also confirmed by Western blot analysis (Figure 4C), as MNU induced suppression of CDK2/cyclin A expression by the overexpression of p21. These expression changes were reversed by CA-HE50, and significant changes were observed only with the expression of p21 and CDK2 (Figure 4D,E) (*p* < 0.05). CA-HE50 tended to increase the expression of cyclin A but not at a level of significance (Figure 4F).

### 3.4. Asiaticoside, a Functional Component of CA-HE50, Inhibits MNU-induced Apoptosis

**Figure 5:**
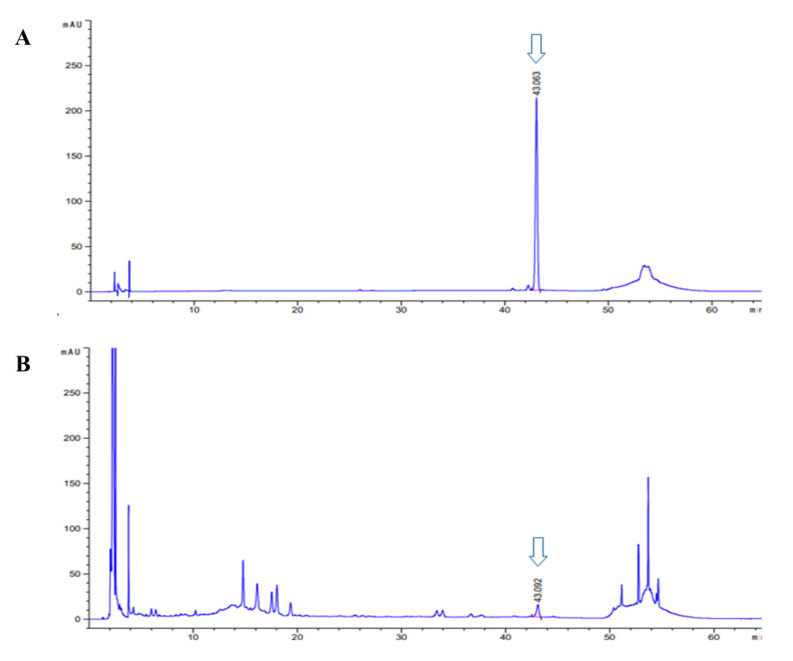
High-performance liquid chromatography (HPLC-PDA) chromatogram of asiaticoside and CA-HE50. HPLC chromatogram of: (**A**) asiaticoside (standard); and (**B**) CA-HE50.

**Figure 6:**
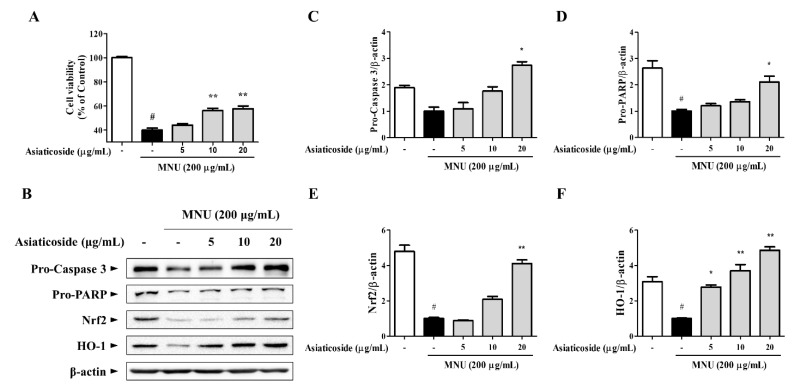
The anti-apoptotic efficacy of CA-HE50 by the activated antioxidant signaling pathway is attributed to asiaticoside. (**A**) Cytoprotective effect of asiaticoside against MNU. (**B**) Western blot analysis for expression of proteins associated with retinal damage with MNU. Asiaticoside efficacy results on: (**C**) pro-caspase-3 protein expression; (**D**) pro-PARP protein expression; (**E**) Nrf2 protein expression; and (**F**) HO-1 protein expression. ^#^ *p* < 0.05 vs. normal control group. * *p* < 0.05 and ** *p* < 0.01 vs. MNU-treated group. Abbreviations: MNU, *N*-methyl-*N*-nitrosourea; PARP, poly (ADP-ribose) polymerase; Nrf2, nuclear factor erythroid 2; HO-1, heme oxygenase-1.

We conducted a study to determine the anti-apoptotic efficacy of asiaticoside, which was identified as a functional substance of CA-HE50 in a previous study [19]. We confirmed the presence of asiaticoside in CA-HE50, and the results are shown in Figure 5. Asiaticoside was detected at 43.0 min and had a content of 14.1 ± 0.03 mg/g in CA-HE50. Asiaticoside showed similar effects as CA-HE50 and inhibited MNU-induced cytotoxicity in a concentration-dependent manner (*p* < 0.01) (Figure 6A). These results were found to be due to an increase in the pro-form expression of caspase-3 and PARP through activation of the Nrf2/HO-1 signaling pathway by asiaticoside (Figure 6B–F). As shown in Figure 6C,D, pro-caspase 3 was detected at higher levels in response to 20 μg/mL asiaticoside compared with controls, and pro-caspase-3 and pro-PARP showed statistically significant differences in cells treated with a high dose of asiaticoside (*p* < 0.05). In addition, as shown in Figure 6E,F, the expressions of the Nrf2/HO-1 antioxidant pathway factors were dramatically increased by asiaticoside, and HO-1 was expressed more strongly at 10 μg/mL asiaticoside compared with controls (*p* < 0.05).

### 3.5. A2E Oxidation Inhibition and Cell Protection Effects of CA-HE50 and Asiaticoside

**Figure 7:**
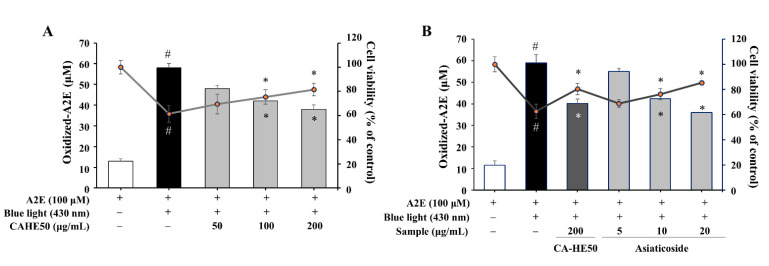
CA-HE50 and asiaticoside exhibit cytoprotective effects by inhibiting the accumulation of oxidized A2E in ARPE-19 cells through antioxidant activity. The cytoprotective efficacy of (**A**) CA-HE50 and (**B**) asiaticoside by the inhibition of oxidized-A2E production and accumulation. ^#^ *p* < 0.05 vs. normal control group. * *p* < 0.05 vs. A2E-treated group. Abbreviations: A2E, *N*-retinylidene-*N*-retinylethanolamine.

A2E, a metabolic by-product of RPE cells, induces dysfunction and apoptosis by accumulating and oxidizing in cells [21,22]. Therefore, we induced A2E oxidation by blue light (430 nm) in ARPE-19 cells and examined the antioxidant effect and cytoprotective effects of CA-HE50 and asiaticoside. CA-HE50 inhibited the A2E oxidation caused by blue light and showed an effect in cell protection (*p* < 0.05) (Figure 7A). As shown in Figure 7B, asiaticoside was also confirmed to be effective in inhibiting the oxidation of A2E and exerting cell protection (*p* < 0.05), which may be due to the antioxidant function of asiaticoside.

## 4. Discussion

AMD shows increasing prevalence and incidence rates worldwide, and the risk of vision loss is very high, especially in late AMD. Currently, various therapeutic methods, including laser therapy, antibody injection, and steroid injection, have been developed for late AMD treatment, which play an important role in preventing vision loss [23,24,25]. However, since treatments to restore retinas to pre-macular degeneration states have not yet been developed, suppressing macular degeneration or inhibiting progression from early AMD to late AMD is more important in preventing vision loss and is the subject of intensive research [21]. We discovered a new natural product that could prevent the occurrence of AMD in a previous study [19], and we conducted the current study to further evaluate the efficacy of the natural product extracts and their underlying mechanisms.

Ocular RPE cells are damaged through various pathways, such as apoptosis, cell cycle arrest, cell death, oxidative damage, and inflammation, due to UVB irradiation and oxygen use [22]. In addition, stress caused by light performs oxidative phosphorylation (OxPhos) of the retinal rod outer segment (OS) disk film without mitochondria and generates reactive oxygen intermediates (ROI), causing damage and loss to the OS of the rod cell [26,27]. To protect against such damage, cells use an antioxidant defense system to negate damage and increase viability. In particular, it was confirmed that metformin and resveratrol can weaken the ROI generation and OxPhos rate of OS through an antioxidant mechanism against oxidative damage and loss [27]. Therefore, the normalization of photoreceptor thickness identified in our study was predicted to be due to the antioxidant efficacy of CA-HE50 against OxPhos-induced damage. AMD is characterized by reduced photoreceptor cells and retinal pigment epithelium (RPE) dysfunction and is associated with apoptosis of RPE, photoreceptors, and inner nuclear layer cells [28]. The junction of RPE and inner/outer segments refers to the retinal epithelial cells from the border of the inner and outer nodes of the photoreceptor and is an important factor in the prognosis of vision recovery in macular diseases [29,30]. Therefore, protecting the cell layers such as macular photoreceptors, RPE, and ONL from damage plays an important role in maintaining vision and preventing vision damage. Our experimental results show that CA-HE50 is directly involved in the inhibition of AMD progression. The decrease in the number of cells and cell layer thickness is controlled by apoptosis and cell cycle progression, which determine cell survival. Therefore, we verified the effect of CA-HE50 on two signaling pathways: the apoptosis and cell cycle pathways. The pathways that induce apoptosis are divided into the extrinsic pathway and the intrinsic pathway. Through these two apoptosis pathways, caspase-3 is cleaved and transformed into an active form. Cleaved caspase-3 generates cleavage of pro-form PARP in the nucleus after nuclear import and induces DNA fragmentation and chromatin condensation to cause cell death [31,32]. The extrinsic apoptosis pathway induces apoptosis by binding stimuli such as TNF-α, FasL, and TRAIL to the death receptor and activating caspase-3 and -7 through activation of caspase-8 and -10. The intrinsic apoptosis pathway induces mitochondria damage by activation of Bax/Bak of the BCL-2 family by stimuli such as DNA damage, mitotic damage, ER stress, oncogene activation, nutritional stress, and hypoxia. As a result, caspase-9 is activated by the apoptosome and then caspase-3 and -7 are activated, leading to apoptosis. In addition, tBid, which is activated by activated caspase-8 and -10 in the extrinsic apoptosis pathway, also activates the intrinsic apoptosis pathway [33]. We measured caspase-8 and -9 to determine whether CA-HE50 affects the extrinsic and intrinsic apoptosis pathways. Caspase-3 and PARP were measured to confirm the effect of CA-HE50 on the expression of final factors leading to apoptosis in vitro study. In addition, in an in vivo study, all ocular tissues were homogenized to confirm the expression level of caspase-3. Our results show that the anti-apoptotic effects of CA-HE50 was found to occur by its significantly inhibiting the intrinsic apoptosis pathway and directly inhibiting the activation of caspase-3 and PARP, which induces apoptosis.

Meanwhile, the cell cycle, another pathway for regulating cell survival, is regulated by multiple control points at several stages, the three principal ones being G1/S, G2/M, and metaphase/anaphase transmission during mitosis. Failure to regulate cell cycle control points leads to abnormal cell growth or apoptosis [34]. Cell senescence and cell cycle arrest caused by various stressors are important factors in aging-related diseases including AMD [35]. Cell cycle arrest, increased apoptosis, and significantly reduced ocular cell layer thickness by MNU in our study support this claim. Therefore, CA-HE50, which suppresses apoptosis and normalizes the cell cycle, shows important promise for preventing AMD symptoms, and this effect was judged to be due to its antioxidant activity.

Many previous studies have confirmed that the Nrf2/HO-1 signaling pathway protects RPE cells by playing a critical role in the development of AMD by oxidative damage [36,37]. Similarly, in our study, CA-HE50 was found to protect ARPE-19 cells through the Nrf2/HO-1 signaling pathway. In addition, this antioxidant effect helps the cell survival of APRE-19 cells by inhibiting the oxidation of A2E and inhibiting its accumulation in cells. Studies have shown that the Nrf2/HO-1 pathway prevents cell cycle arrest by inhibiting p21 expression caused by cell damage signals [38]. Likewise, in our study, suppression of p21 expression affects the expression of cdk2 and cyclin A, and, consequently, the cell cycle seems to have been normalized.

## 5. Conclusions

We confirmed the mechanism of CA-HE50 in preventing AMD progression. In our previous studies, we showed that CA-HE50 inhibited the activation of caspase-3 and PARP and increased the expression of Nrf2/HO-1. In the present study, we comprehensively analyzed the effect of CA-HE50 on the survival of cells through various experiments (verification of extrinsic/intrinsic apoptosis signaling pathway, measurement of cell cycle arrest, and evaluation of antioxidant signaling pathways). Our results determine that the cytoprotective effect of CA-HE50 in APRE-19 cells and animal experiment occurred through the inhibition of the intrinsic apoptosis signaling pathway and prevention of cell cycle arrest caused by activation of the Nrf2/HO-1 signaling pathway. In addition, our data further confirmed that this strong antioxidant effect is due to the asiaticoside present in CA-HE50. These findings demonstrate the potent antioxidant-induced cytoprotective effects of CA-HE50 and asiaticoside in inhibiting ARPE-19 cell and macular degeneration at both in vitro and in vivo levels. In addition, it was confirmed that there was no toxicity in the preclinical safety evaluation including genotoxicity (not published). These results confirm the possibility of developing CA-HE50 as a functional raw material for eye health and suggest the necessity of a human application test using CA-HE50.

## Data Availability

Data is contained within the article.

## References

[1] Friedman D.S., O’Colmain B.J., Muñoz B., Tomany S.C., Mccarty C., De Jong P.T.V.M., Nemesure B., Mitchell P., Kempen J. (2004). Prevalence of age-related macular degeneration in the United States. Arch. Ophthalmol..

[2] Coleman H.R., Chan C.-C., Ferris F.L., Chew E.Y. (2008). Age-related macular degeneration. Lancet.

[3] Wong W.L., Su X., Li B.X., Cheung C.M.G., Klein B.E., Cheng C.-Y., Wong T.Y. (2014). Global prevalence of age-related macular degeneration and disease burden projection for 2020 and 2040: A systematic review and meta-analysis. Lancet Glob. Health.

[4] Evans J.R. (2001). Risk factors for age-related macular degeneration. Prog. Retin. Eye Res..

[5] Seddon J.M., Sharma S., Adelman R.A. (2006). Evaluation of the clinical age-related maculopathy staging System. Ophthalmology.

[6] Kanagasingam Y., Bhuiyan A., Abràmoff M.D., Smith R.T., Goldschmidt L., Wong T.Y. (2014). Progress on retinal image analysis for age related macular degeneration. Prog. Retin. Eye Res..

[7] Brown C.N., Green B.D., Thompson R.B., Hollander A.I.D., Lengyel I., on behalf of the EYE-RISK Consortium Metabolomics and age-related macular degeneration (2019). Metabolomics and Age-Related Macular Degeneration. Metabolites.

[8] Birben E., Sahiner U.M., Sackesen C., Erzurum S., Kalayci O. (2012). Oxidative stress and antioxidant defense. World Allergy Organ. J..

[9] Bellezza I., Mierla A.L., Minelli A. (2010). Nrf2 and NF-κB and their concerted modulation in cancer pathogenesis and progression. Cancers.

[10] Zhao Z., Chen Y., Wang J., Sternberg P., Freeman M.L., Grossniklaus H.E., Cai J. (2011). Age-related retinopathy in NRF2-deficient mice. PLoS ONE.

[11] Gao X., Talalay P. (2004). Induction of phase 2 genes by sulforaphane protects retinal pigment epithelial cells against photooxidative damage. Proc. Natl. Acad. Sci. USA.

[12] Dulull N.K., Dias D.A., Thrimawithana T.R., Kwa F.A.A. (2018). L-Sulforaphane confers protection against oxidative stress in an in vitro model of age-related macular degeneration. Curr. Mol. Pharmacol..

[13] Frede K., Ebert F., Kipp A.P., Schwerdtle T., Baldermann S. (2017). Lutein activates the transcription factor Nrf2 in human retinal pigment epithelial cells. J. Agric. Food Chem..

[14] Orhan C., Akdemir F., Tuzcu M., Sahin N., Yilmaz I., Ali S., Deshpande J., Juturu V., Sahin K. (2016). Mesozeaxanthin protects retina from oxidative stress in a rat model. J. Ocul. Pharmacol. Ther..

[15] Evans J.R., Lawrenson J.G. (2017). Antioxidant vitamin and mineral supplements for preventing age-related macular degeneration. Cochrane Database Syst. Rev..

[16] Lawrenson J.G., Evans J.R. (2015). Omega 3 fatty acids for preventing or slowing the progression of age-related macular degeneration. Cochrane Database Syst. Rev..

[17] Satia J.A., Littman A.J., Slatore C.G., Galanko J.A., White E. (2009). Long-term use of beta-carotene, retinol, lycopene, and lutein supplements and lung cancer risk: Results from the VITamins And Lifestyle (VITAL) study. Am. J. Epidemiol..

[18] Jamil S.S., Nizami Q., Salam M. (2007). *Centella asiatica* (Linn.) Urban—A Review. Nat. Prod. Radiance.

[19] Park D.W., Jeon H., So R., Kang S.C. (2020). *Centella asiatica* extract prevents visual impairment by promoting the production of rhodopsin in the retina. Nutr. Res. Pract..

[20] Maurer E., Tschopp M., Tappeiner C., Sallin P., Jaźwińska A., Enzmann V. (2014). Methylnitrosourea (MNU)-induced retinal degeneration and regeneration in the zebrafish: Histological and functional characteristics. J. Vis. Exp..

[21] SanGiovanni J.P., Chew E.Y., Clemons T.E., Rd F.F., Gensler G., Lindblad A.S., Milton R.C., Seddon J.M., Sperduto R.D., Age-Related Eye Disease Study Research Group (2007). The relationship of dietary carotenoid and vitamin A, E, and C intake with age-related macular degeneration in a case-control study: AREDS Report No. 22. Arch. Ophthalmol..

[22] Mahendra C.K., Tan L.T.H., Pusparajah P., Htar T.T., Chuah L.-H., Lee V.S., Low L.E., Tang S.Y., Chan K.-G., Goh B.H. (2020). Detrimental Effects of UVB on Retinal Pigment Epithelial Cells and Its Role in Age-Related Macular Degeneration. Oxidative Med. Cell. Longev..

[23] Canton V.M., Quiroz-Mercado H., Velez-Montoya R., Lopez-Miranda M.J., Moshfeghi A.A., Shusterman E.M., Kaiser P.K., Sanislo S.R., Gertner M., Moshfeghi D.M. (2011). 16-Gy Low-Voltage X-ray Irradiation with Ranibizumab Therapy for AMD: 6-Month Safety and Functional Outcomes. Ophthalmic Surg. Lasers Imaging Retin..

[24] Augustin A. (2009). Triple therapy for age-related macular degeneration. Retina.

[25] Machida S., Nishimura T., Harada T., Kurosaka D. (2012). Retinal ganglion cell function after repeated intravitreal injections of ranibizumab in patients with age-related macular degeneration. Clin. Ophthalmol..

[26] Bruschi M., Bartolucci M., Petretto A., Calzia D., Caicci F., Manni L., Traverso C.E., Candiano G., Panfoli I. (2020). Differential expression of the five redox complexes in the retinal mitochondria or rod outer segment disks is consistent with their different functionality. FASEB J..

[27] Calzia D., Degan P., Caicci F., Bruschi M., Manni L., Ramenghi L.A., Candiano G., Traverso C.E., Panfoli I. (2018). Modulation of the rod outer segment aerobic metabolism diminishes the production of radicals due to light absorption. Free Radic. Biol. Med..

[28] Dunaief J.L., Dentchev T., Ying G.-S., Milam A.H. (2002). The role of apoptosis in age-related macular degeneration. Arch. Ophthalmol..

[29] Frenkel S., Hendler K., Siegal T., Shalom E., Pe’Er J. (2008). Intravitreal methotrexate for treating vitreoretinal lymphoma: 10 years of experience. Br. J. Ophthalmol..

[30] Chan A., Duker J.S., Ishikawa H., Ko T.H., Schuman J.S., Fujimoto J.G. (2006). Quantification of photoreceptor layer thickness in normal eyes using optical coherence tomography. Retina.

[31] Igney F.H., Krammer P.H. (2002). Death and anti-death: Tumour resistance to apoptosis. Nat. Rev. Cancer.

[32] Elmore S. (2007). Apoptosis: A review of programmed cell death. Toxicol. Pathol..

[33] Kalkavan H., Green D.R. (2018). MOMP, cell suicide as a BCL-2 family business. Cell Death Differ..

[34] Barnum K.J., O’Connell M.J. (2014). Cell cycle regulation by checkpoints. Methods Mol. Biol..

[35] Blasiak J., Piechota M., Pawlowska E., Szatkowska M., Sikora E., Kaarniranta K. (2017). Cellular Senescence in Age-Related Macular Degeneration: Can Autophagy and DNA Damage Response Play a Role?. Oxidative Med. Cell. Longev..

[36] Clementi M.E., Sampaolese B., Sciandra F., Tringali G. (2020). Punicalagin Protects Human Retinal Pigment Epithelium Cells from Ultraviolet Radiation-Induced Oxidative Damage by Activating Nrf2/HO-1 Signaling Pathway and Reducing Apoptosis. Antioxidants.

[37] Zhu C., Dong Y., Liu H., Ren H., Cui Z. (2017). Hesperetin protects against H_2_O_2_-triggered oxidative damage via upregulation of the Keap1-Nrf2/HO-1 signal pathway in ARPE-19 cells. Biomed. Pharmacother..

[38] Villeneuve N.F., Sun Z., Chen W., Zhang D.D. (2009). Nrf2 and p21 regulate the fine balance between life and death by controlling ROS levels. Cell Cycle.

